# Palatine tonsillar metastasis of rectal adenocarcinoma: a case report and literature review

**DOI:** 10.1186/1477-7819-11-114

**Published:** 2013-05-25

**Authors:** Hao Wang, Ping Chen

**Affiliations:** 1 Department of Gastrointestinal Surgery, Northern Jiangsu People’s Hospital, Yangzhou University, Yangzhou, Jiangsu Province, 225001, PR China

**Keywords:** Tonsil neoplasm, Metastasis, Rectal neoplasm, Adenocarcinoma, Immunohistochemistry

## Abstract

Cases of primary colorectal adenocarcinoma metastasized to the palatine tonsil are extremely rare. To the best of our knowledge, only 10 cases have thus far been previously documented in the English literature. A 37-year-old Chinese woman presented with a right palatine tonsil swelling and odynophagia 5 months after a surgical resection of rectal adenocarcinoma was performed. The patient underwent a tonsillectomy, and a metastatic poorly differentiated adenocarcinoma from a colorectal origin was revealed by immunohistochemical analysis. The manner in which tonsillar metastases are involved remains unknown and should be further studied. Here, we report a new case, briefly summarize these 10 cases and review the literature.

## Background

The most common sites of distant metastases from primary colorectal carcinoma are in the liver, lung, and brain, and less commonly in the bone, ovary, and adrenal gland. Metastasis to palatine tonsil from a primary colorectal carcinoma is an extremely rare event. Only 10 cases have thus far been previously documented in the English literature. Hematogenous dissemination is a probable explanation for the mechanism of metastasis to the palatine tonsils [1], as well as the suggestion of a retrograde cervical lymphatic spread through the thoracic duct [2].

A metastatic tumor in an unusual site may sometimes be troublesome to distinguish between a synchronous or metachronous primary cancer and a metastatic disease, especially when it is asymptomatic. In this paper, we report the case of a 37-year-old Chinese woman with a metastasis to the right palatine tonsil from a rectal adenocarcinoma and review the literature.

## Case presentation

A 37-year-old Chinese woman was evaluated for right tonsil swelling and a sore throat in our hospital. She was diagnosed in September 2011 with rectal cancer revealed by generalized peritonitis evoked by tumor perforation. At diagnosis, the preoperative evaluation did not show distant metastasis (M0). The patient underwent an urgent exploratory laparotomy. The tumor measuring 10.0 cm×4.0 cm×3.5 cm was identified at the anterior rectal wall under the peritoneal reflection intraoperatively. A low anterior resection using a total mesorectal excision technique was performed, with a colostomy using noninflamed descending colon, and the divided end of the rectum was closed. The excised specimen contained a poorly differentiated adenocarcinoma invading into nonperitonealized perirectal fat with negative surgical margins (T3). Nine regional lymph nodes were positive for tumor extension (N2b). The tumor was classified as stage IIIc disease based on the American Joint Committee on Cancer TNM staging system.

**Figure 1:**
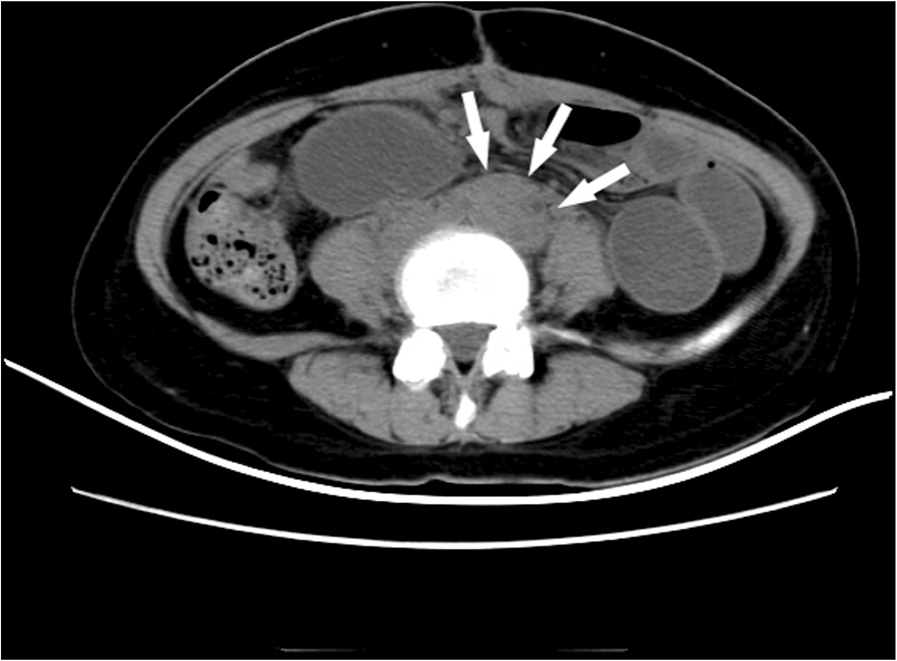
**Nonenhanced abdominal computed tomography scan. **The scan revealed para-aortic lymph node enlargement measuring approximately 4 cm in diameter (arrows).

**Figure 2:**
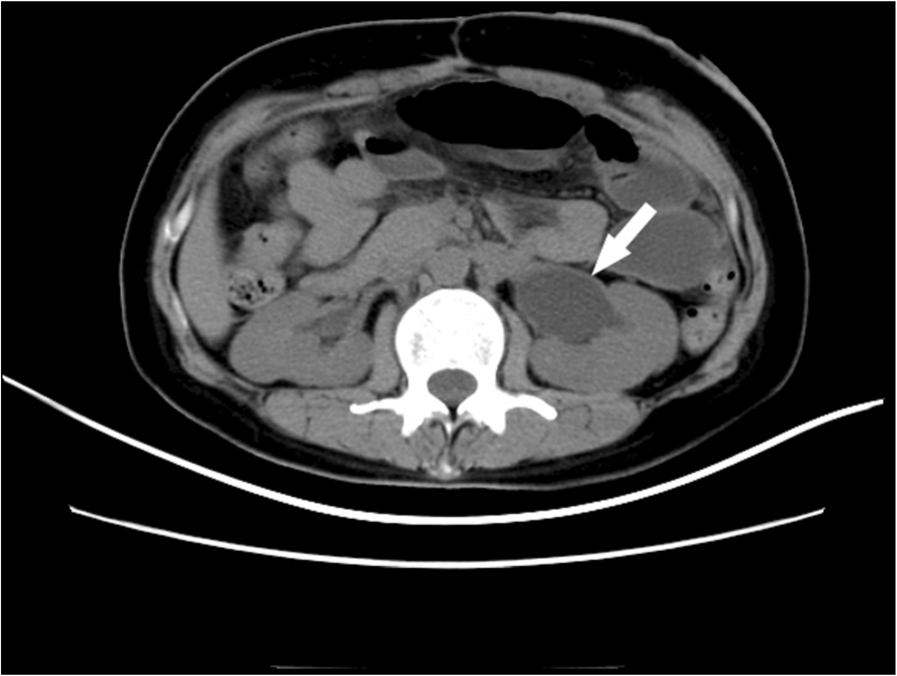
**Enlarged para-aortic lymph nodes compressed the left upper ureter causing left ureteral obstruction and hydronephrosis. **Arrow, left ureteral obstruction.

The patient did not receive any adjuvant radiotherapy and chemotherapy postoperatively. In March 2012 the patient was admitted to our department on account of abdominal pain and distension triggered by small bowel obstruction. A nonenhanced abdominal computed tomography scan revealed enlarged para-aortic lymph nodes measuring approximately 4 cm in diameter (Figure 1), which compressed the left upper ureter (Figure 2). No evidence of cerebral and visceral metastasis or mediastinal lymph nodes was identified by computed tomography scan of the brain, chest, and abdomen.

**Figure 3:**
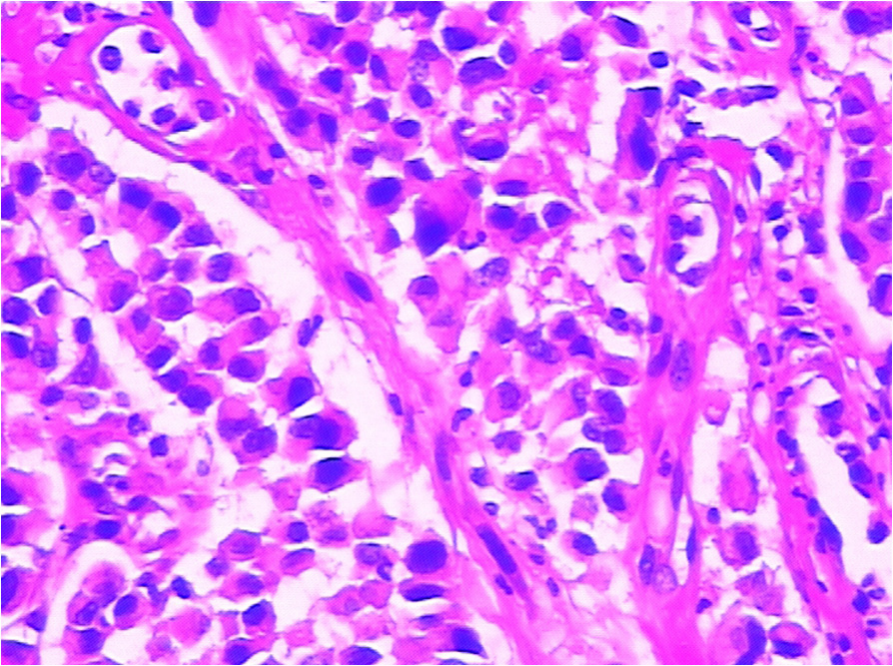
**Microscopic section of the biopsy tissue showed abundant heterogenic cells with a neoplastic gland formation. **H & E, magnification ×200.

**Figure 4:**
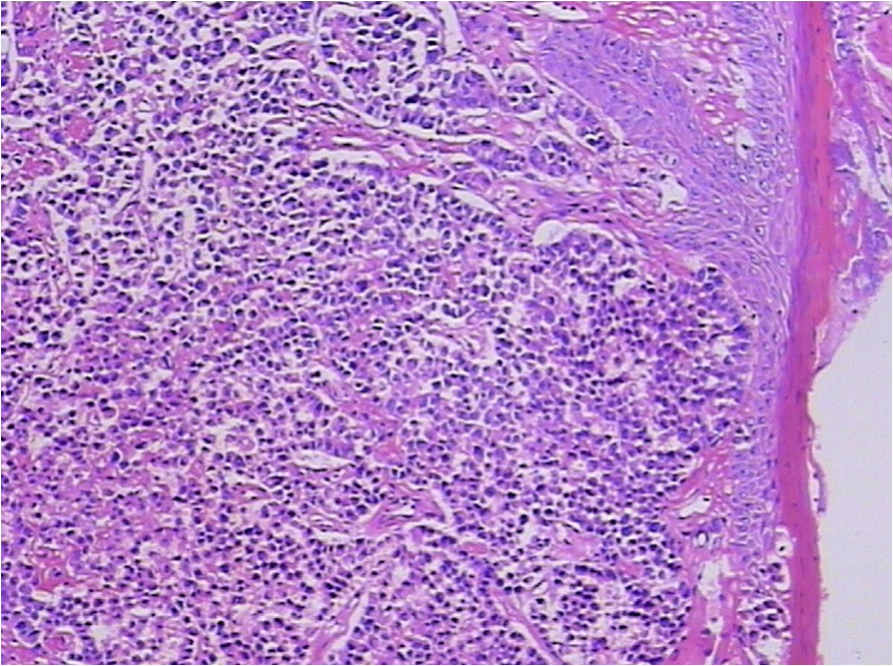
**Histopathological examination of the resected tonsillar specimen. **Histopathology showed surface squamous epithelium with extensive infiltration of the tonsillar lamina propria by abundant malignant small glandular cells. H & E, magnification ×100.

**Figure 5:**
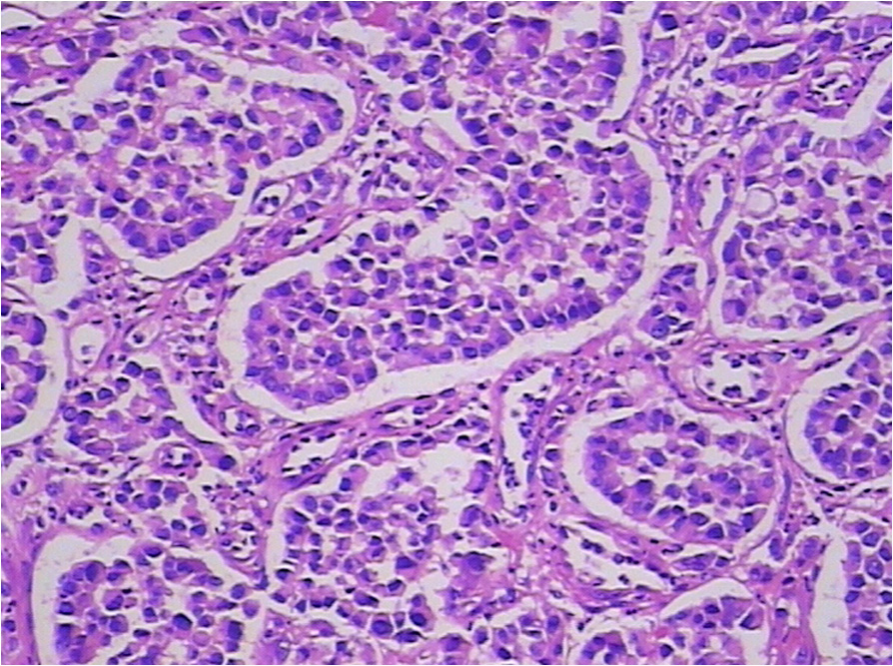
**Histopathological feature of the tonsillar tumor: abundant malignant small glandular cells. **H & E, magnification ×200.

In the following days, the patient complained of swelling of her right tonsil and odynophagia. She did not report symptoms of dysphagia or tonsillitis. Physical examination revealed an ulcerated mass measuring about 1 cm in maximum diameter, on the upper part of her right palatine tonsil. There were no palpable cervical lymph nodes. The rest of the physical examination was unremarkable. Routine laboratory parameters including the complete blood count, erythrocyte sedimentation rate, C-reactive protein level, liver and pancreas enzymes, and tumor markers (carcinoembryonic antigen, carcinoma antigen 125, carcinoma antigen 199, and α-fetoprotein) were all within normal limits. A punch biopsy was taken for histological examination, which showed a poorly differentiated adenocarcinoma (Figure 3). The patient underwent a palliative right tonsillectomy without neck dissection. Microscopic examination of the resected specimen disclosed surface squamous epithelium with extensive infiltration of the tonsillar lamina propria by abundant malignant small glandular cells (Figures 4 and 5). Immunohistochemical analysis results of tumor cells are presented in Table 1. These features confirmed the diagnosis of metastatic poorly differentiated adenocarcinoma of the right palatine tonsil identical to the colorectal primary. At the time of submission of the present manuscript the patient was still alive, 9 months after the diagnosis of metastatic disease.

**Table 1:** Immunohistochemical analysis results of palatine tonsillar tumor cells

**Antibody**	**P/N**	**Antibody**	**P/N**	**Antibody**	**P/N**
bcl-6	–	CD56	–	EMA	+++
CD1a	–	CD79a	–	Granzyme B	–
CD10	–	CDX-2	+++	mum-1	–
CD138	+	CK20	++	p63	–
CD20	–	CK34βE12	–	Perforin	–
CD3	–	CK5/6	–	S100	–
CD45-LCA	–	CK7	–	TdT	–
CD45Ro	–	CKpan	++	villin	+++

P/N positive/negative, CK cytokeratin, EMA epithelial membrane antigen, mum-1, melanoma-associated antigen (mutated) 1 TdT terminal deoxynucleotidyl transferase, –, no cells positive by immunohistochemistry (IHC); ±, sometimes weak positive, sometimes negative by IHC; +, <25% of cells positive by IHC; ++, 25 to 50% of cells positive by IHC; +++, >50% of cells positive by IHC.

## Discussion

Metastases to tonsils from nonhematological malignant neoplasms are rare events [3], accounting for only 0.8% of all tonsillar malignancies [4]. Malignant melanoma [5], renal cell carcinoma [2], breast carcinoma [6], and lung carcinoma [7] have been described as the most common primaries of tonsillar metastases. Adenocarcinoma of the stomach [8] and carcinoma of the pancreas [9] and seminomas [10] are less common primary sites. Sporadic cases of tonsillar metastasis have been reported from prostate carcinoma [11], gall bladder carcinoma [12], anaplastic thyroid carcinoma [13], Merkel cell carcinoma [14], choriocarcinoma [15], and malignant mesothelioma [16].

Metastasis from a primary colorectal adenocarcinoma to the palatine tonsil is an extremely rare event. We searched PubMed, MEDLINE, and Google Scholar, from inception to December 2012, using the terms ‘colorectal/colon/colonic/rectum/rectal’; ‘cancer/ carcinoma/adenocarcinoma’; ‘palatine tonsil/tonsil’; and ‘metastasis’. The literature was limited to English-language case reports. References of included articles were also searched. Only 10 cases have been documented previously. We present a summary of all these 10 cases, as well as the present case, to highlight their clinicopathological profiles (Table 2). In a total of 11 patients, the age ranged from 36 to 81 years (mean: 53.5 years; median: 53 years), having a male-to-female ratio of 1.75:1 (7 vs. 4). In our case, the patient was a 37-year-old woman with a primary rectal adenocarcinoma that had metastasized to the right palatine tonsil. This is the youngest female patient to be reported. The metastases to palatine tonsils have a tendency to manifest unilaterally, while the left side (7/11) was more commonly involved than the right (4/11). Involvement of both sides was not observed. Contradictorily, it had been reported that malignant melanoma metastatic to the tonsil usually manifests bilaterally [2,8]. Of the 11 cases, seven patients had enlarged cervical lymph nodes when the palatine tonsil mass was found, while seven patients had primary lesions with metastatic regional lymph nodes, and one patient had metastatic evidence in the liver, two patients in the lung, three patients in the brain, three patients in bone, two patients in the mediastinum, two patients in the subcutis, and one patient in the axilla. Metastatic palatine tonsillar adenocarcinoma is a systematic malignancy that harbors a poor prognosis irrespective of the differentiation of the primary tumor and stage of the disease. Even though only 10 cases have been reported, the life expectancy ranged from 6 to 15 months, no matter whether the patient is treated by palliative chemoradiotherapy or tonsillectomy.

**Table 2:** Clinicopathological features of reported cases of metastatic palatine tonsil tumor of colorectal primary

**Case (reference)**	**Sex/age (years)**	**Side**	**Primary site**	**Differentiation**	**Stage**	**Interval (months)**	**Other metastases**	**Follow-up (months)**
1 [17]	F/55	Right	Rectum	Well	NA	84	Mediastinum	NA
2 [18]	M/65	Left	Transverse colon	Poorly	NA	0	Para-aortic LN, bone, scalp	6
3 [19]	M/36	Right	Rectum	Signet-ring cell	Dukes C	24	NA	15 alive
4 [20]	F/81	Left	Hepatic flexure	Moderately	NA	0	Lung, liver, bone	12
5 [21]	M/53	Right	Rectum	Poorly	Dukes C2	24	Brain	6 alive
6 [22]	M/45	Left	Rectum	Signet-ring cell	NA	0	Subcutaneous, bone	6
7 [23]	M/44	Left	Cecum	Signet-ring cell	NA	0	NA	NA
8 [24]	M/53	Left	Ascending colon	Moderately	T3N1M0	19	Brain	13 alive
9 [1]	F/76	Left	Splenic flexure	Signet-ring cell	T3N2M0	12	Brain, right axilla	NA
10 [25]	M/43	Left	Left colon	Moderately	T4aN2bM0	12	Lung, mediastinum	NA
Present case	F/37	Right	Rectum	Poorly	T3N2bM0	5	Para-aortic LN	9 alive

F female, M male, Well, well differentiated adenocarcinoma; moderately, moderately differentiated adenocarcinoma; poorly, poorly differentiated adenocarcinoma; Signet-ring cell, signet-ring cell carcinoma; interval, time between the diagnosis of colorectal carcinoma and the development of metastasis to the palatine tonsil; LN lymph nodes, NA not available.

In the metastatic process, tonsillar involvement could either be the first station or a part of widespread systematic distant metastases. Although the pathway by which malignancies metastasized to the tonsil remains controversial and difficult to determine, some hypotheses have been built. Brownson and colleagues suggested that retrograde cervical lymphatic spread through the thoracic duct may be a potential mechanism, since the palatine tonsil does not have afferent lymphatic vessels [2]. On the other hand, hematogenous spread to the tonsil may occur through the systematic arterial blood flow passing through the lungs. Or tumor cells can reach the brain or head and neck region bypassing the lungs via venous blood flow through Batson’s plexus [1]. In the present case, evidence of metastases to the liver, lungs, brain, and bone were not observed and no cervical lymphadenopathy was palpated –metastasis to the unilateral palatine tonsil through Batson’s plexus may therefore be a more reasonable explanation.

**Figure 6:**
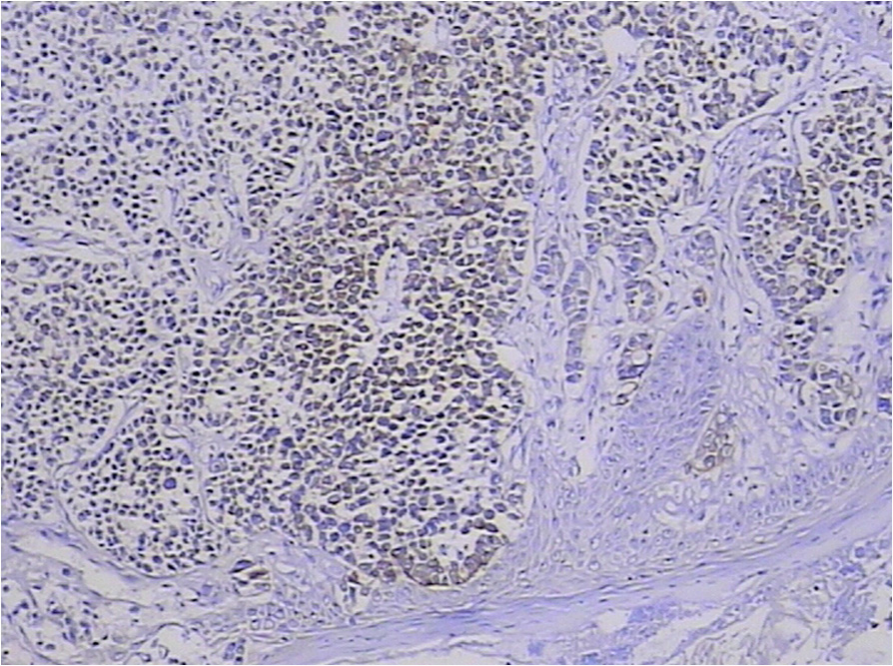
**Tumor cytoplasm was cytokeratin 20-positive.** 3,3′-Diaminobenzidine, magnification ×100.

**Figure 7:**
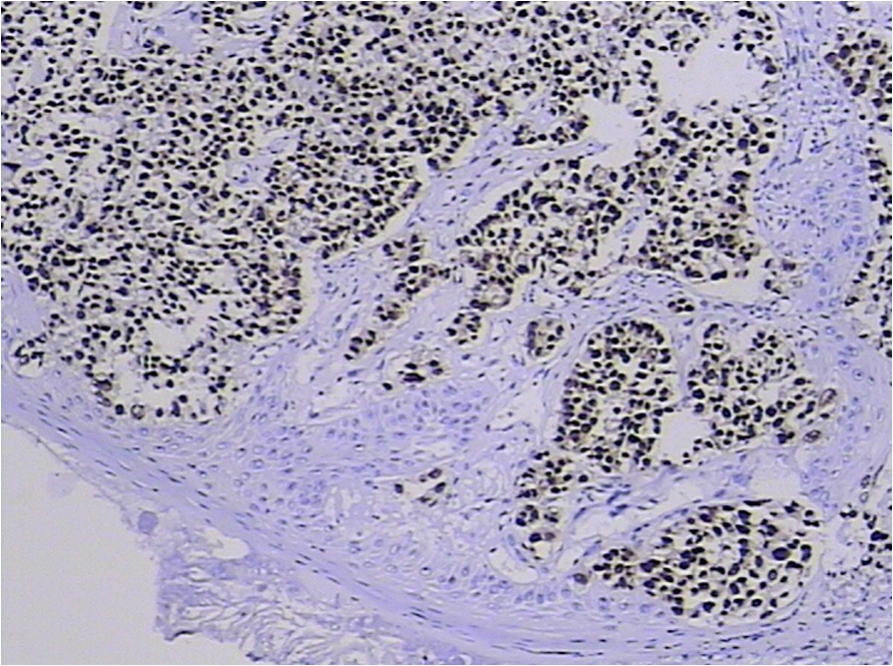
**Tumor cell nucleus was positive for CDX-2.** 3,3′-Diaminobenzidine, magnification ×100.

**Figure 8:**
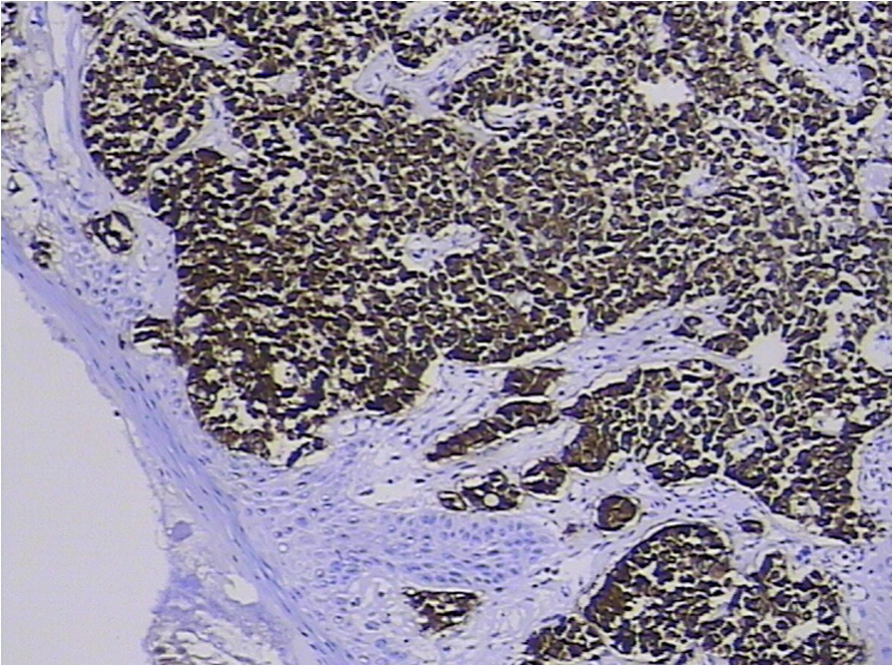
**Homogeneous diffuse membrane and cytoplasm uptake of anti-villin antibodies in tumor cells.** 3,3′-Diaminobenzidine, magnification ×100.

**Figure 9:**
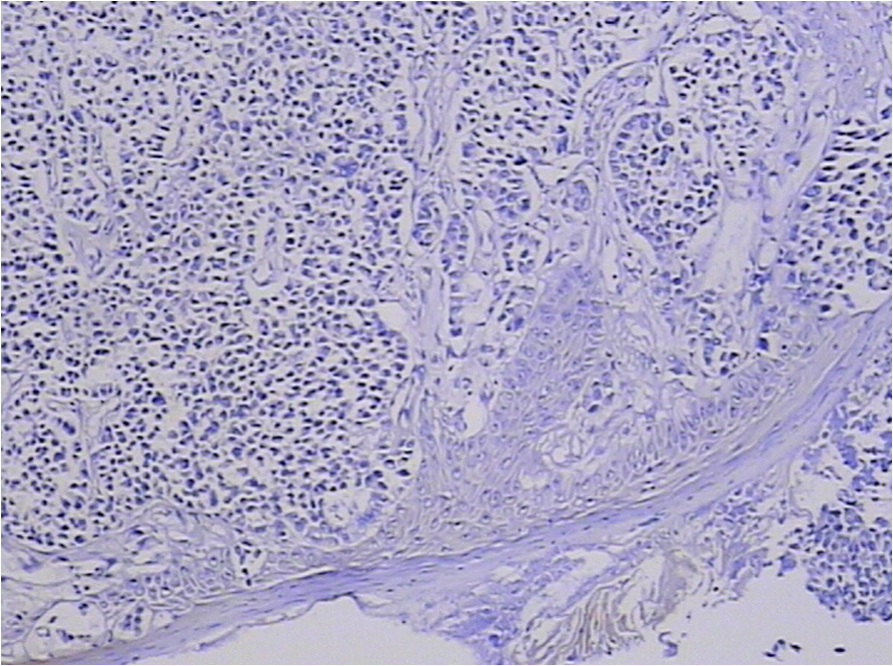
**Tumor cells were negative for cytokeratin 7.** 3,3′-Diaminobenzidine, magnification ×100.

Malignancies of the palatine tonsil are unusual. Squamous epithelial carcinomas and lymphomas are generally observed in this area. In the present case, the palatine tonsillar tumor cells shown a glandular epithelial phenotype histologically (Figure 5), and were negative for both squamous epithelial carcinoma markers [cytokeratin (CK) 34βE12, CK5/6, and p63] and lymphoma markers [bcl-6, CD1a, CD10, CD20, CD3, CD45-LCA, CD45Ro, CD56, CD79a, granzyme B, melanoma-associated antigen (mutated) 1, perforin, and terminal deoxynucleotidyl transferase] immunohistochemically (Table 1). Since there is no glandular epithelium in the palatine tonsil, a metastatic adenocarcinoma should be incorporated into the differential diagnosis. Adenocarcinoma of unknown primary often occurs in clinical practice. Even in the era of advanced imaging techniques and molecular tests, identification of the site of origin for metastatic adenocarcinoma frequently poses a challenge to clinicians and pathologists, and may lead to different therapeutic consequences. Immunohistochemical analysis remains a mainstay choice in identifying the histological origin of palatine tonsillar tumor with an occult primary. Although only few of tumor markers are very specific and have high sensitivity, several markers with moderate specificity are available, and when used in panels the discriminating capacity of these markers may be sufficient. The different expression patterns of CK20, CK7, CDX-2 and villin can be useful [26,27]. Given that 78% of adenocarcinomas of the upper gastrointestinal tract express both CK20 and CK7, most colorectal adenocarcinomas are positive for CK20 but negative for CK7 [26,27]. Further, metastatic lung adenocarcinoma shows a respiratory-type phenotype (CK20–/CK7+/ CDX-2–/villin–), while metastatic colorectal adenocarcinoma shows an intestinal-type phenotype (CK20+/CK7–/CDX-2+/villin+, as in our case; Figures 6, 7, 8 and 9) [28].

## Conclusions

Metastatic palatine tonsil cancer from a primary colorectal adenocarcinoma is an extremely rare malignancy with a poor prognosis, and may lay a pitfall for clinicians. Immunohistochemical examination should therefore be performed. Immunomarkers including CK20, CK7, CDX-2, and villin are facilities in immunohistochemistry examination. The route of metastasis to the tonsil remains unclear and should been studied further.

## Consent

Written informed consent was obtained from the patient for publication of this case report and any accompanying images. A copy of the written consent is available for review by the Editor-in-Chief of this journal.

## Abbreviations

CK: Cytokeratin; H & E: Hematoxylin and eosin.

## Competing interests

The authors declare that they have no competing interests.

## Authors’ contributions

HW performed the majority of this study and drafted the manuscript. PC provided the collection of material from the database. All authors read and approved the final manuscript.
